# The Dismissed Paris Nurses

**Published:** 1888-02-25

**Authors:** 


### THE EDITOR'S LETTER-BOX.

[Our Correspondents are reminded that prolixity is a great bar to publication, and that brevity of style and conciseness of statement greatly facilitate early publication.]

### THE DISMISSED PARIS NURSES.

It may interest those who have to do with hospital administration to read the following letter, addressed by the chief members of the medical staff of the great hospital of La Charité in Paris to the Lady Superior of the nursing Sisters of the hospital, who have been expelled in consequence of a vote of the Paris Municipality. The article from which the letter is extracted says that the Augustinian Sisters left la Charité on January 25th last, and that if the same deference is paid to a recent resolution of the Municipal Council, they will, in the course of the year, leave the hospitals of St. Louis and the Hôtel Dieu. They are to be replaced by lay nurses who have had only a year or so of insufficient training, and who will cost more than treble the amount of the Sisters. There is probably something of weight to be put forward on the other side, but it is very clear that the medical officers, who should be good judges of the qualities of a nurse, are bitterly opposed to the change. C.

"Madam,—Before the departure of the Augustinian Sisters so unjustly dismissed from the Hospital of La Charité, and because the governing body, whom they have so long and loyally served, does not deem it its duty to address to them a word of thanks, be pleased to accept here the expression of our gratitude towards the most excellent nuns whom our patients are about to lose. All that we have attempted in order to preserve for our hospital the Augustinian Sisters is in vain; but they will at least carry away with them this memory, that the physicians and surgeons of the hospital have not abandoned those who always seconded them to the utmost. This testimony of justice which we render to them will soften for ourselves the bitterness of our helplessness. We desire to say no more. The life of the hospital nun is above all praise. Hospital Sisters will be for long years yet, among all the nations on earth, the purest expression of devotion and of sacrifice.—Receive, madam, the assurance of our high consideration.

(Signed)

"Potain, Desnos, Fereol, Luys, Laboullière, and Blachez, Physicians to the Hospital; Trélat and Després, Surgeons to La Charité."

